# Serum Expression of miR-23a-3p and miR-424-5p Indicate Specific Polycystic Ovary Syndrome Phenotypes: A Pilot Study

**DOI:** 10.3390/ijms25063205

**Published:** 2024-03-11

**Authors:** Olivia Trummer, Jonas Hoeller, Sharmaine Reintar, Veronika Tandl, Ines Foessl, Valentin Borzan, Verena Theiler-Schwetz, Christian Trummer, Elisabeth Lerchbaum, Barbara Obermayer-Pietsch

**Affiliations:** 1 Department of Internal Medicine, Division of Endocrinology and Diabetology, Medical University of Graz, 8036 Graz, Austria; 2 Graz Ragnitz Private Hospital, 8047 Graz, Austria

**Keywords:** miRNA, polycystic ovary syndrome, PCOS phenotypes, Rotterdam criteria, hyperandrogenism

## Abstract

MicroRNAs (miRNAs) are single-stranded, non-coding RNAs that regulate mRNA expression on a post-transcriptional level. Observational studies suggest an association of serum miRNAs and polycystic ovary syndrome (PCOS), a common heterogeneous endocrinopathy characterized by hyperandrogenism (HA), oligo- or amenorrhea (OM) and polycystic ovaries. It is not known whether these miRNA profiles also differ between PCOS phenotypes. In this pilot study, we compared serum expression profiles between the four PCOS phenotypes (A–D) and analyzed them both in PCOS (all phenotypes) and in phenotypes with HA by quantitative-real-time PCR (qRT-PCR). The serum expression of miR-23a-3p was upregulated in phenotype B (*n* = 10) and discriminated it from phenotypes A (*n* = 11), C (*n* = 11) and D (*n* = 11, AUC = 0.837; 95%CI, 0.706–0.968; *p* = 0.006). The expression of miR-424-5p was downregulated in phenotype C (*n* = 11) and discriminated it from phenotypes A, B and D (AUC = 0.801; 95%CI, 0.591–1.000; *p* = 0.007). MiR-93-5p expression was downregulated in women with PCOS (all phenotypes, *n* = 42) compared to controls (*n* = 8; *p* = 0.042). Phenotypes with HA (A, B, C; *n* = 32) did not show differences in the analyzed expression pattern. Our data provide new insights into phenotype-specific miRNA alterations in the serum of women with PCOS. Understanding the differential hormonal and miRNA profiles across PCOS phenotypes is important to improve the pathophysiological understanding of PCOS heterogeneity.

## 1. Introduction

Polycystic ovary syndrome (PCOS) is a common endocrine condition in women of reproductive age, affecting between 6 and 22% of all women world-wide, depending on the definition [1,2]. Since the establishment of the Rotterdam consensus in 2003, PCOS has been defined by the presence of at least two out of three criteria: clinical and/or biochemical hyperandrogenism (HA), oligo- or amenorrhea (OM) and/or polycystic ovarian morphology (PCOM). In addition to these three main characteristics, many women have several other comorbidities or consecutive diseases, such as disorders of glucose and lipid metabolism as well as low-grade inflammation [2,3]. Women with PCOS also have a higher prevalence of infertility, pregnancy complications, depression, obesity, type 2 diabetes, iron overload [4] and the related long-term outcomes [2,5]. The pathogenesis of PCOS has not been fully understood, but it is assumed to be multifactorial, comprising genetics, the intrauterine environment, the microbiome and lifestyle factors such as diet, exercise or stress [2,6].

In PCOS, four phenotypes were defined according to certain constellations of the Rotterdam criteria: phenotype A (HA, OM, PCOM), phenotype B (HA, OM), phenotype C (HA, PCOM) and phenotype D (OM, PCOM). Many studies have shown that PCOS symptom severity as well as insulin resistance and other comorbidities occur mostly in women with phenotypes with HA (A, B, C), while phenotype D shows a milder form of PCOS [7,8,9].

HA has a composite origin, attributed mainly to the ovaries, with a substantial contribution from the adrenals and a minor contribution from adipose tissue [10]. It is now widely recognized that insulin resistance, manifesting above all in obese or overweight women but often also in lean women with PCOS, is one of the keys to this complex syndrome [11,12]. Increased levels of insulin through insulin resistance determines HA by acting synergically with luteinizing hormone (LH) on ovarian steroidogenic enzymes. Since the enzymes involved in ovarian steroidogenesis are similar to those of the adrenal glands, many studies have shown that insulin may act directly as a stimulator of adrenal steroidogenesis [13,14]. Furthermore, insulin also contributes to HA by inhibiting sex hormone binding globulin (SHBG) production by the liver [15,16].

MicroRNAs (MiRNAs) are small non-coding RNAs that regulate gene expression at a post-transcriptional level by either suppressing translation or inducing mRNA degradation [17]. These RNA molecules are transcribed from intergenic genomic sequences or intronic regions of protein-coding genes [18]. Individual miRNAs can target up to 100 different mRNAs, potentially influencing the expression of entire gene networks. In total, over 30% of human mRNAs are regulated by miRNAs [17]. Mature miRNAs can be released as extracellular miRNAs into various body fluids, such as blood, serum, plasma or follicular fluid, and the dysregulation of these circulating miRNAs is observed in various diseases and physiological states [19]. Research indicates that levels of circulating miRNAs play an important role in the progression of metabolic diseases [20], malignancies [21] and endocrine system disorders [22]. Several studies have shown that women with PCOS show altered miRNA expression profiles in different cell types and body fluids [23,24,25,26] such as the ovary [27], follicular fluid [28], blood [29], serum [24] and adipocytes [30]. Since extracellular miRNAs are stable in serum, easy to detect and potentially disease-specific, they became ideal biomarkers for various diseases and conditions [31] including PCOS [32,33,34]. Data on miRNA expression in PCOS phenotypes [25,26,35] are scarce and have not yet been compared in serum between all four PCOS phenotypes according to the Rotterdam classification. The main aim of this pilot study was to compare the serum expression profiles of 12 miRNAs previously related with PCOS between the four PCOS phenotypes and to analyze them, both as PCOS-specific and in phenotypes with HA.

## 2. Results

### 2.1. Clinical Characterization

We included a total of 51 participants in our study. Of these, 11 were diagnosed with PCOS phenotypes A, C and D, respectively, and 10 with phenotype B. Eight women who did not fulfil any of the Rotterdam criteria were classified as controls. The baseline characteristics of all selected participants are shown in Table 1.

#### 2.1.1. PCOS in Comparison to Controls

Women with PCOS (all phenotypes) showed significantly higher serum levels of anti-Müllerian hormone (AMH, *p* < 0.001), luteinizing hormone (LH, *p* = 0.031), the LH/follicle stimulating hormone (FSH) ratio (*p* = 0.046), androstenedione (*p* < 0.001), total testosterone (TT, *p* < 0.001) and free testosterone (fTesto, *p* < 0.001). The Ferriman–Gallwey score (FGS) was more than twice as high in women with PCOS (7.79 ± 7.14) as in the control group (3.25 ± 4.5, *p* = 0.002). Age and body mass index (BMI) were balanced between the groups.

#### 2.1.2. Phenotype-Specific Comparison to Controls

Concentrations of androstenedione (*p* = 0.031), TT (*p* < 0.001) and fTesto (*p* < 0.001) were significantly higher in women with phenotype A.

Compared to controls, women with phenotype B showed higher levels of AMH (*p* = 0.043), androstenedione (*p* < 0.001) and TT (*p* = 0.001).

In women with phenotype C, serum levels of dehydroepiandrosterone (DHEA, *p* = 0.033), TT (*p* = 0.024) and FTesto (*p* = 0.011) were increased. Compared to the controls, the FGS was more than three times higher (*p* = 0.010).

Phenotype D showed no significant differences compared to the control group.

### 2.2. Basic Expression of miRNA Candidates

We evaluated the basic expression of our miRNA candidates according to their amount of expression and categorized them into three groups:

- “Not detectable”

In our study cohort, miR-592 and miR-6767-5p were not expressed at all.

- “Expression at the detection limit”

**Figure 1:**
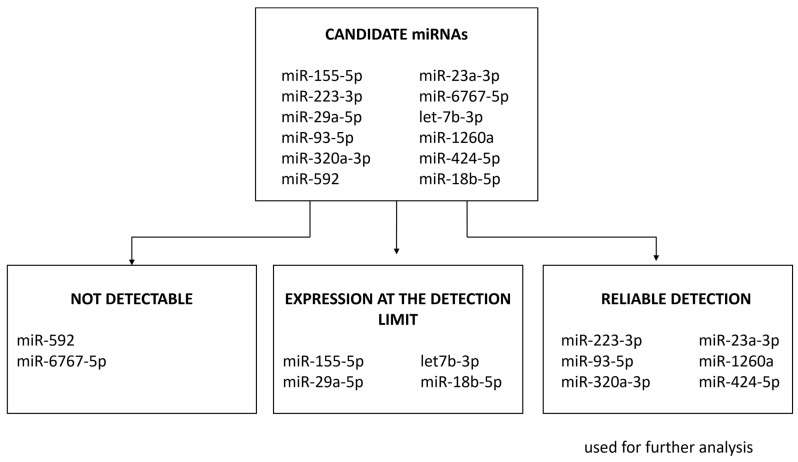
Evaluation of the miRNA candidate expression in the study cohort.

For miR-155-5p, miR-29a-5p, let-7b-3p and miR-18b-5p, the miRNA concentration in the investigated samples was too low to be reliably measured with our method. Some samples showed specific amplification curves that did not reach the plateau phase within the 40 predefined cycles, indicating extremely low expression. For the remaining samples, the miRNA concentration was below the detection limit. These samples were considered as “undetectable” (Figure 1). MiRNAs of this group showed high proportions of undetectable samples [miR-155-5p: *n* = 37 (72.5%), miR-29a-5p: *n* = 30 (58.8%), let-7b-3p: *n* = 24 (47.1%) and miR-18b-5p: *n* = 31 (60.8%)] and were therefore excluded from the statistical group comparisons.

- “Reliable detection of expression”.

MiR-223-3p, miR-93-5p, miR-320a-3p, miR-23a-3p, miR-1260a and miR-424-5p showed valid amplification and melting curves within an acceptable ΔCq range between duplicates. These miRNAs were further analyzed.

### 2.3. Phenotype-Specific miRNA Expression

**Figure 2:**
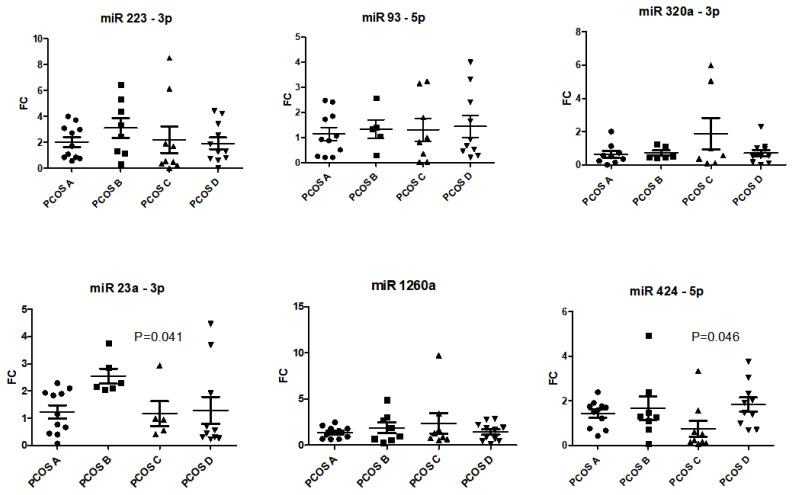
Serum miRNA expressions in samples of PCOS phenotypes A, B, C and D. Data are displayed as scatter plots, where each dot represent the fold change as 2^−ΔΔCq^ value of one study sample. Significance was tested by Kruskal–Wallis test.

MiR-23a-3p and miR-424-5p showed altered serum expressions in one phenotype compared to the other phenotypes. MiR-23a-3p was significantly upregulated in phenotype B compared to phenotypes A, C and D. MiR-424-5p was significantly downregulated in phenotype C compared to phenotypes A, B and D (Table 2 and Figure 2).

**Figure 3:**
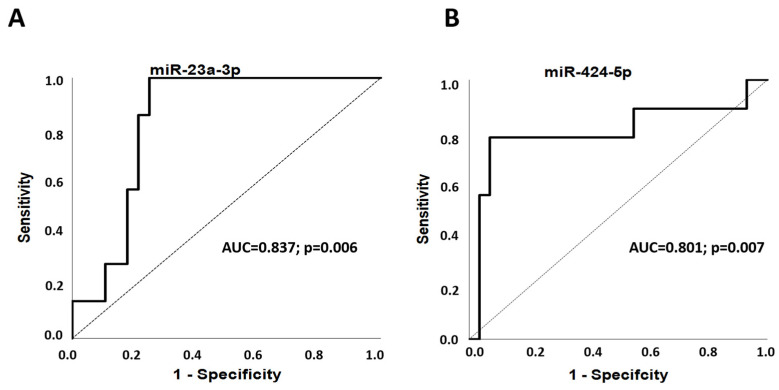
The discriminatory potential of the serum expressions of miR-23a-3p and miR-424-5p in the prediction of PCOS phenotypes displayed in ROC curves. (**A**) The discriminatory potential of miR-23a-3p serum expression to differentiate phenotype B from A, C and D. (**B**) The discriminatory potential of miR-424-5p serum expression to differentiate phenotype C from A, B and D.

To evaluate the discriminatory potential of miR-23a-3p and miR-424-5p serum expressions in predicting a PCOS phenotype, we performed receiver-operating characteristic (ROC) analysis and calculated the area under the curve (AUC) value. MiR-23a-3p serum expression is a discriminator to differentiate phenotype B from phenotypes A, C and D (AUC = 0.837; 95% confidence interval (CI), 0.706–0.968; *p* = 0.006). MiR-424-5p discriminates phenotype C from A, B and D (AUC = 0.801; 95%CI, 0.591–1.000; *p* = 0.007) (Figure 3).

The serum expression of miR-223-3p, miR-93-5p, miR-320a-3p, miR-1260a and miR-424-5p showed no group-specific differences between PCOS phenotypes (Table 2).

### 2.4. PCOS-Specific Expression Compared to Controls

**Figure 4:**
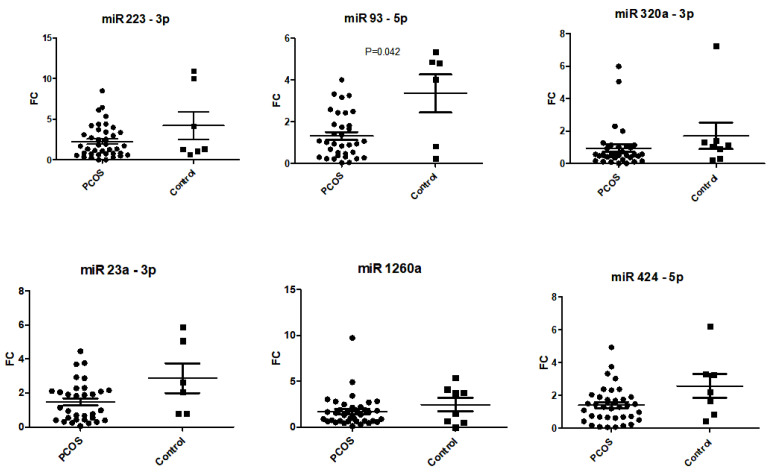
Expression of candidate miRNAs in serum of women with PCOS (all phenotypes) and controls. Data are displayed as scatter plots, where each dot represents the fold change as 2^−ΔΔCq^ of one study sample.

**Figure 5:**
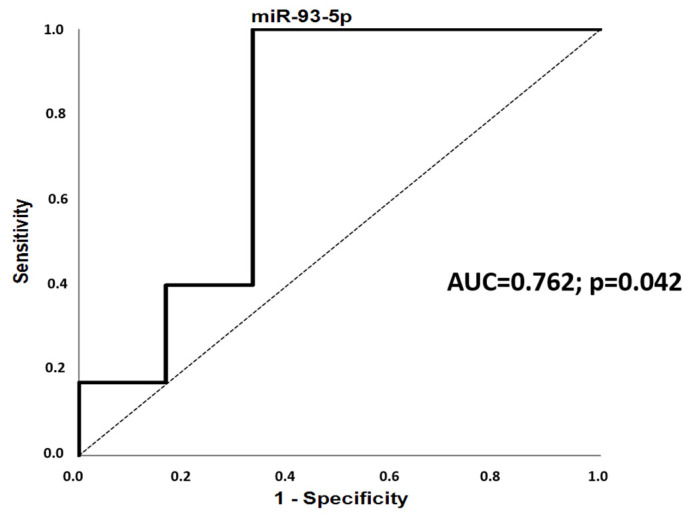
The discriminatory potential of miR-93-5p serum expression to differentiate between PCOS status and controls. ROC, receiver-operating characteristic; AUC, area under the curve; CI, confidence interval.

The serum expression of miR-93-5p was downregulated in women with PCOS (all phenotypes) compared to the control group (*p* = 0.042) (Table 3 and Figure 4). This association did not remain significant after Bonferroni correction for multiple testing. The fold change values of miR-93-5p discriminated women with PCOS from controls (AUC = 0.762; 95%CI, 0.483–1.000; *p* = 0.042) in the ROC analysis (Figure 5). MiR-223, miR-93-5p, miR-320a-3p, miR-23a-3p, miR-1260 and miR-424-5p showed no group-specific differences in their serum expression (Table 3). Respective scatter plots are shown in Figure 4.

### 2.5. Expression in Phenotypes with HA

**Figure 6:**
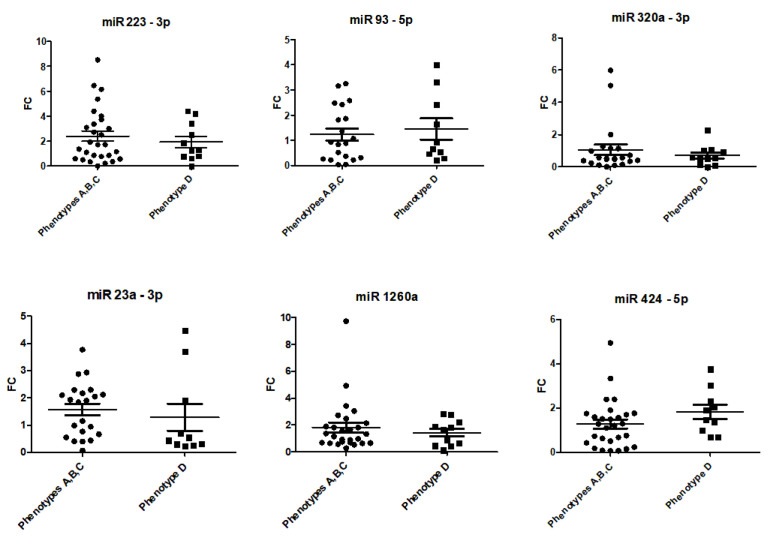
Serum expression of candidate miRNAs in phenotypes with HA (phenotypes A, B, C) and women with PCOS without signs of HA (phenotype D). Data are displayed as scatter plots, where each dot represents the fold change as 2^−ΔΔCq^ of one study sample.

A subgroup analysis between phenotypes with HA (A, B, C; *n* = 32) and women with PCOS without signs of HA (phenotype D, *n* = 11) showed no group-specific differences in their serum miRNA expression profiles. Respective scatter plots are shown in Figure 6.

## 3. Discussion

Between PCOS phenotypes, we observed an upregulated serum expression of miR-23a-3p in phenotype B and a downregulation of miR-424-5p in phenotype C. Within PCOS, the determined serum expression of these miRNAs differentiated the respective phenotype from the other phenotypes. The comparison of serum miRNA expressions between PCOS (all phenotypes, *n* = 43) and controls (*n* = 8) showed miR-93-5p as being downregulated in women with PCOS (all phenotypes, *n* = 43). A subgroup analysis of the phenotypes with HA (*n* = 32) did not show altered miRNA serum profiles compared to phenotype D without HA (*n* = 11).

HA is a key feature of women with PCOS and occurs in three of the four PCOS phenotypes. It is caused by a disruption of normal ovarian or adrenal function, leading to excessive androgen production. Ovarian miRNA expression profiles in tissue [36], granulosa cells [37], cumulus cells [25,38] and follicular fluid [25,38,39] have been shown to be altered in women with PCOS. We therefore assumed that serum miRNA profiles alter between phenotypes with HA and phenotype D (no HA), which was not the case in our data set. We are aware that equal sample sizes are more powerful than unequal sample sizes, and therefore, a possible difference in miRNA expression may not be proven to be significant (type II error) [40]. A recent study demonstrated altered serum expression profiles of miRNAs, including miR-320a-3p, in PCOS phenotypes with HA compared to the control group [26]. In our data, the miR-320a-3p mean expression value of phenotype D (no HA) in serum was even lower compared to phenotypes with HA, although this difference was not significant. We would also like to mention a study by Motahari et al. that describes differences in serum miRNAs (other than those described here) between three of the four Rotterdam PCOS phenotypes (A: *n* = 8; C: *n* = 5; and D: *n* = 6) [25].

Our results support several mechanistic studies that relate the differentially expressed miRNAs to the pathophysiology of PCOS.

MiR-23a-3p: Wei et al. demonstrated in their study that an overexpression of miR-23a-3p negatively targets the high-mobility group at hook 2 (HMGA2) to block the Wnt/β-catenin signaling pathway, thereby suppressing viability and promoting apoptosis in granulosa cells [41] in women with PCOS who have lower apoptosis rates of granulosa cells in their ovaries [42]. Compared to the other phenotypes, we observed a significant overexpression of miR-23a-3p in the serum of phenotype B, which would be consistent with the defined characteristic of OM in phenotype B and the mechanism suggested by Wei et al. [41].

MiR-424-5p: Exosomal miR-424-5p derived from the follicular fluid of women with PCOS inhibits granulosa cell proliferation and induces cellular senescence in PCOS by blocking cell division cycle-associated 4 (CDCA4)-mediated Rb/E2F1 signaling. This mechanism, again, may contribute to abnormal follicular development in women with PCOS [43]. Compared to the other phenotypes, we found miR-424-5p to be downregulated in the serum of women with phenotype C, which is in line with the PCOM features of this phenotype.

MiR-93-5p: Jiang et al. published that the increased expression of mir-93 in granulosa cells promoted granulosa cell proliferation by targeting cyclin-dependent kinase inhibitor 1A (CDKN1A) [44]. Accordingly, Tan and colleagues demonstrated that the overexpression of miR-93-5p promotes apoptosis and ferroptosis in granulosa cells of women with PCOS by regulating the NF-kB signaling pathway [45]. Silencing miR-93-5p protected against granulosa cell dysfunction. MiR-93-5p was found to be upregulated in PCOS, both in granulosa cells [35,44,46] as well as in serum [46,47], which is not consistent to our observations in the serum, where miR-93-5p expression was significantly downregulated compared to the control group. Possible reasons for varying results in miRNA studies are discussed in detail in a later section of the discussion.

The identified miRNAs, miR-23a-3p, miR-424-5p and miR-93-5p, should be considered as potential therapeutic targets for improving granulosa cell functions in women with PCOS and need to be further investigated in future research studies.

Our study design broke down PCOS heterogeneity into its defined phenotypes. We compared the serum expressions of miRNAs previously related to women with PCOS without further phenotype classification between the four phenotypes of PCOS. Although it was not our primary aim, we also calculated fold change values of miRNAs in all women with PCOS (all phenotypes, *n* = 44) and compared them to controls (*n* = 8). We only replicated one miRNA, miR-93-5p, out of six valid miRNA candidates that was also downregulated in PCOS (Sathyapalan et al. [46]). In this analysis, too, we cannot rule out a type II error due to the unequal sample size. The reproducibility of data between miRNA studies is a challenge. To enable accurate data comparison between studies, pre-analytical steps such as blood drawing and serum/plasma preparation and analysis and evaluation methods, as well as the reporting, need to be standardized.

In the case of miRNAs, very low or no concentrations from circulation might be expected [48]. The handling of missing qPCR values from qPCR is therefore another critical point that requires standardization. Failure to distinguish between missing data due to a low concentration or missing data due to randomly occurring technical errors may further partly explain the variation within and between otherwise similar studies. To improve the accuracy and precision of our miRNA data, we applied an adopted practical data handling pipeline [48] and evaluated the investigated candidate miRNAs according to the level of expression into the categories “not detectable”, “at the detection limit” and “reliable detection” (Figure 1). Data reproducibility of miRNA studies is essential prior to clinical applications [49].

There are further, more specific reasons for the distinct results of this study regarding the non-replicated miRNA candidates, especially miR-592 and miR-6767-5p, which were not detected at all in our cohort. Inter-ethnic expression differences between Asian (Han Chinese: Song J. et al. [50], Xiong et al. [51], Zhang C. et al. [37], Ding et al. [52]; Korean: Song D. et al. [53]), Egyptian (Rashad et al. [54]) and Caucasian (present study) cohorts could contribute to these varying outcomes. We speculate that miR-592 and miR-6767-5p are not expressed in the serum of Caucasian women. Butler et al. [39] reported altered expressions of miR-1260, miR-424-5p, miR-let7b-3p and miR-18b-5p in the plasma of women with PCOS that we did not observe in serum. The latter two miRNAs showed expressions at the detection limit in our cohort. Serum and plasma differ in their content of miRNA derived from different blood cells [55,56,57], which may explain these incongruent findings. However, there are further inconsistencies for miR-155-5p and miR-29a-5p (both show expression at the detection limit in our data) that cannot be explained by differences in demographic diversity, analytical method or sample type. Random biological variability or the influence of unknown confounder variables between sample cohorts cannot be ruled out and could be responsible for these contradictory data, highlighting the challenges of data reproducibility in miRNA studies.

The results of the present study are, as already mentioned, limited by the relatively small number of participants per group or within a group and should therefore be investigated in more detail in larger cohorts of PCOS phenotypes. Furthermore, we cannot estimate how the investigated miRNAs vary in their expressions before, during or after the development of PCOS phenotypes, since this study design was observational after developing the current phenotype. During the course of PCOS, the phenotype can also develop into a different phenotype. We can also not distinguish between causal miRNAs and the affected ones from the global pool of miRNAs that are implicated in PCOS phenotypes.

Among the strengths of our study are the in-depth clinical and biochemical characterization of all women with PCOS; the breakdown of PCOS heterogeneity into four age- and BMI-matched PCOS phenotypes; and the application of a data handling pipeline to assure statistical validity and improve reproducibility.

In summary, we provide new data on phenotype-specific miRNA alterations in women with PCOS. The miRNAs miR-23a-3p and miR-424-5p, detected in serum, are indicators for phenotype B, with a higher severity, and phenotype C, a milder type of PCOS. Insights into miRNA profiles across PCOS phenotypes contribute to the pathophysiological understanding of the heterogeneity of PCOS and may be important for future research in personalized medicine as well as diagnostic and treatment strategies for women with PCOS.

## 4. Materials and Methods

### 4.1. Study Design

This pilot study was designed to investigate miRNA profiles from sources within the literature on premenopausal women affected by four different PCOS phenotypes.

### 4.2. Study Population

The samples of the present study were age- and BMI-matched from a previously described cross-sectional PCOS study [7,58,59]. In brief, women visited the outpatient clinic of the Department of Endocrinology and Diabetology, Department of Internal Medicine, Medical University of Graz, for PCOS-related symptoms such as hirsutism, acne, PCOM or menstrual irregularities, or were recruited by expert staff such as medical doctors or study nurses. After the exclusion of related disorders with similar clinical features, PCOS was diagnosed according to the Rotterdam criteria if two out of the following three characteristics were met: clinical and/or biochemical signs of HA, OM and/or PCOM [60].

Clinical HA was defined as the presence of hirsutism [modified FGS > 4] [61], acne, alopecia and/or seborrhea. Biochemical HA was defined by elevated TT (>0.77 ng/mL), fTesto (>3.18 pg/mL), androstenedione (>3.2 ng/mL) and dehydroepiandrosterone sulfate (DHEA-S > 2.75 ng/mL). OM was diagnosed with a mean cycle length >35 or <25 days for the past 12 months or a single cycle >90 days. PCOM was established via transvaginal ultrasound (TVU) by experienced gynecologists [7].

Detailed patient histories were taken, and physical examinations were performed to determine height, weight and clinical signs of HA.

Women with PCOS were phenotyped using the following constellations of the Rotterdam criteria: phenotype A (HA, OM and PCOM), phenotype B (HA, OM), phenotype C (HA, PCOM) and phenotype D (OM, PCOM) [7].

Women of the control group were premenopausal and hormonally healthy and showed no Rotterdam criterion [7].

All study participants were at least 18 years old and provided written informed consent. This study was conducted in compliance with the Declaration of Helsinki and approved by the ethics committee of the Medical University of Graz (EK 18-066 ex 06/07).

### 4.3. Laboratory Measurements

Blood samples for the measurement of hormones were taken after an overnight fast. Serum AMH levels were measured by immunoassay (Beckmann Coulter, Krefeld, Germany). The levels of LH and FSH were determined using Access hLH and hFSH CLIA (Beckman Coulter, Brea, CA, USA), while sex hormone binding globulin (SHBG) was assessed using Elecsys ECLIA (Roche Diagnostics, Mannheim, Germany). DHEA-S was determined by an ELISA (Labor Diagnostika Nord, Nordhorn, Germany). Androstenedione was measured by using IMMULITE CLIA assays (Siemens Healthcare Diagnostics Products Ltd., Glyn Rhonwy, UK), while TT was examined by ADVIA Centaur Immunoassays (Siemens Healthcare Diagnostics Inc., Tarrytown, NY, USA). FTesto was measured using the ACTIVE Free Testosterone Radioimmunoassay (Immunotech s.r.o., Prague, Czech Republic). The summary of all assay reference ranges as well as intra- and inter-assay coefficients of variance have been previously published [7].

### 4.4. Selection of miRNAs

Candidate miRNAs for the present investigation were selected according to their presence in serum or plasma, as well as to previously described associations with PCOS-related features such as ovarian follicle growth, insulin resistance [42,47], free androgen index (FAI) [62] or number of menses per year [52]. These miRNAs and their literature sources are described in Table 4.

### 4.5. MiRNA Isolation and qPCR

MiRNA was isolated using the miRNeasy Serum/Plasma Advanced Kit (Qiagen, Hilden, Germany) according to the manufacturer’s manual, including the addition of spike-in controls (Uni Sp 2,4,5, RNA Spike in Kit for RT, Qiagen, Hilden, Germany). RNA was eluted from the columns by the addition of 20 μL of RNase-free water and stored in the short term at −80 °C. Complementary DNA (cDNA) was generated using a miRCURY LNA RT synthesis kit (Qiagen, Hilden, Germany) including the addition of Uni Sp6. Subsequent (semi)quantitative real-time PCR (qPCR) was performed using a miRCURY LNA SYBR Green PCR Kit and specific miRCURY LNA miRNA PCR Assays (both from Qiagen, Hilden, Germany) with the CFX384 Touch Real-Time PCR Detection System (Bio-Rad, Hercules, CA, USA). All qPCRs were conducted in duplicates, with inter-plate calibration and a maximum of 40 cycles.

### 4.6. qPCR Data Analysis

Background levels for each miRNA assay were assessed using a nuclease-free water sample. The quality of extraction, cDNA synthesis and qPCR was checked using synthetic spike-in controls during miRNA isolation (Uni Sp2,4,5), cDNA synthesis (Uni Sp6) and PCR amplification (cel-miR-39-3p). Cycle of quantification (Cq) values were determined using the Cq regression determination mode. The average of the Cq values for each duplicate was calculated. The acceptable Cq range of duplicates was defined as the interval in which 95% of the Cq values are expected to be found, given a certain Cq value [48]. We selected the highest Cq representing the lowest observed miRNA input that yielded an analyzable result, and substituted all undetectable results for each measured miRNA with Cq + 1 [48]. The relative expression levels of all investigated miRNAs were calculated as the fold change [66]. For that, average Cq values were normalized to the mean of snU6 and miR-484, used as endogenous control [67], to calculate Δ (delta) Cq values. The fold change was calculated as 2^−ΔΔCq^, where ΔΔCq was the ΔCq of PCOS phenotypes minus the ΔCq of controls. Quantitative qPCR data are reported as mean ± standard deviation (SD).

### 4.7. Statistical Analysis

A statistical analysis was performed using SPSS Statistics version 28/29 (IBM SPSS Statistics GmbH, Ehringen, Germany). Patient characteristics are reported as mean ± SD unless otherwise stated. The distribution of data was analyzed by descriptive statistics and the Kolmogorov–Smirnov test, as well as by evaluation of quantile–quantile plots. The homogeneity of variances was tested using Levene’s test. Normally distributed quantitative data with equal variances were compared with an ANCOVA or Mann–Whitney U test; unequally distributed data or data with heterogeneous variances were compared with Kruskal–Wallis tests for non-parametric samples. Tukey’s and Dunnet’s post hoc tests were applied for multiple comparisons between the phenotype group and the control group. The diagnostic value for discriminating a PCOS phenotype or PCOS status was assessed by calculating the area under the curve (AUC). Receiver-operating characteristic (ROC) curves were generated by plotting sensitivity versus (1-specificity). A *p*-value of ≤0.05 was considered statistically significant.

## Figures and Tables

**Table 1:** Clinical characterization of the study cohort. Frequency data are presented as numbers, with continuous data as mean ± standard deviation. Normally distributed data were compared using ANCOVA or Mann–Whitney U test. Non-parametric data were compared using the Kruskal–Wallis Test. PCOS, polycystic ovary syndrome; BMI, body mass index; AMH, anti-Mullerian hormone; LH, luteinizing hormone; FSH, follicle-stimulating hormone; SHBG, sex hormone binding globulin; DHEA, dehydroepiandrosterone; TT, total testosterone; fTesto; free testosterone; FGS, modified Ferriman–Gallwey score; PCOM, polycystic ovarian morphology.

Characteristics	Control	PCOS A	PCOS B	PCOS C	PCOS D	PCOS (All Phenotypes)
Number	8	11	10	11	11	43
BMI (kg/m^2^)	24.53 (±4.50)	25.41 (±6.43)	25.09 (±5.64)	26.278 (±7.07)	27.12 (±8.02)	26.00 (±6.67)
Age (yr)	29.15 (±3.57)	26.42 (±3.86)	29.74 (±3.91)	27.61 (±3.63)	27.51 (±3.05)	27.78 (±3.69)
AMH (ng/mL)	3.95 (±1.85)	12.73 (±7.85)	13.04 (±6.59) ^b^	7.18 (±6.03)	9.96 (±5.63)	10.57 (±6.70) ^d^
LH (mIU/mL)	5.34 (±4.37)	11.32 (±7.14)	15.05 (±11.09)	9.45 (±8.78)	8.70 (±5.01)	11.04 (±8.30) ^c^
FSH (mIU/mL)	5.99 (±2.61)	7.01 (±1.97)	6.66 (±1.65)	5.78 (±3.14)	5.94 (±1.00)	6.34 (±2.09)
LH/FSH Ratio	0.90 (±0.52)	1.57 (±0.83)	2.27 (±1.41)	1.90 (±2.10)	1.46 (±0.78)	1.79 (±1.37) ^c^
SHBG (nmol/L)	74.27 (±37.19)	51.93 (±20.65)	66.70 (±43.09)	59.31 (±44.02)	76.55 (±39.01)	63.55 (±37.55)
DHEA (µg/mL)	1.37 (±0.69)	23.33 (±72.16)	1.82 (±0.76)	2.63 (±0.94) ^a^	1.24 (±0.39)	7.38 (±36.47)
androstenedione (ng/mL)	2.33 (±0.58)	3.78 (±1.21) ^a^	4.68 (±1.72) ^a^	3.26 (±1.14)	2.24 (±0.54)	3.46 (±1.46) ^d^
TT (ng/mL)	0.22 (±0.14)	0.59 (±0.22) ^b^	0.56 (±0.14) ^b^	0.46 (±0.15) ^b^	0.43 (±0.16)	0.51 (±0.03) ^d^
fTesto (pg/mL)	1.19 (±0.44)	2.99 (±1.30) ^b^	2.33 (±1.03)	2.64 (±0.85) ^b^	1.67 (±0.57)	2.41 (±1.06) ^d^
Hirsutism (FGS)	3.25 (±4.5)	6.60 (±5.23)	10.44 (±6.37)	12.18 (±8.10) ^b^	1.11 (±1.45)	7.79 (±7.14) ^d^
PCOM	no	yes	no	yes	yes	no/yes

Superscript letters indicate significant *p*-values: ^a^ *p*-value < 0.05 for the PCOS phenotype group compared with the control group using Dunnet post hoc test; ^b^ *p*-value < 0.05 for the PCOS phenotype group compared with the control group using Tukey post hoc test; ^c^ *p*-value < 0.05 for PCOS compared with the control group using Mann–Whitney U test; ^d^ *p*-value < 0.05 for PCOS compared with the control group using a *t*-test.

**Table 2:** MiRNA fold change values according to expressed miRNA candidates of the respective PCOS phenotypes and their *p*-values. Data are shown as median ± standard error of the mean (SEM).

*miRNA*	*Phenotype A* (*n* = 11)	*Phenotype B* (*n* = 10)	*Phenotype C* (*n* = 11)	*Phenotype D* (*n* = 8)	*p*-*Value*
miR-223-3p	0.51 ± 0.20	0.49 ± 0.16	0.56 ± 0.95	0.55 ± 0.19	0.385
miR-93-5p	0.93 ± 0.26	1.36 ± 0.37	0.92 ± 0.46	0.67 ± 0.41	0.847
miR-320a-3p	0.51 ± 0.20	0.49 ± 0.16	0.56 ± 0.95	0.55 ± 0.19	0.952
**miR-23a-3p**	1.16 ± 0.24	2.24 ± 0.27	0.95 ± 0.45	0.49 ± 0.49	**0.041**
miR-1260a	1.37 ± 0.19	1.43 ± 0.57	1.11 ± 1.10	1.66 ± 0.29	0.978
**miR-424-5p**	1.59 ± 0.18	1.30 ± 0.52	0.24 ± 0.36	1.69 ± 0.32	**0.046**

**Table 3:** MiRNA fold changes according to expressed miRNA candidates in women with PCOS (all phenotypes) and in controls. Data are shown as median ± standard error of the mean (SEM). PCOS, polycystic ovary syndrome.

*miRNA*	*Women with PCOS* (*All Phenotypes*) *n* = 43	*Controls n* = 8	*p*-*Value*
miR-223-3p	1.71 ± 0.32	1.35 ± 1.68	0.335
**miR-93-5p**	0.93 ± 0.18	4.41 ± 0.91	**0.042**
miR-320a-3p	0.52 ± 0.23	1.08 ± 0.80	0.122
miR-23a-3p	0.69 ± 0.15	0.80 ± 0.42	0.940
miR-1260a	1.35 ± 0.27	2.62 ± 0.72	0.434
miR-424-5p	1.16 ± 0.12	0.88 ± 0.40	0.928

**Table 4:** Candidate miRNAs with target sequence and reference.

miRNA	Target Sequence	References
hsa-miR-155-5p	UUAAUGCUAAUCGUGAUAGGGGUU	[23,29]
hsa-miR-223-3p	UGUCAGUUUGUCAAAUACCCCA	[63]
hsa-miR-29a-5p	ACUGAUUUCUUUUGGUGUUCAG	[47,52,64]
hsa-miR-93-5p	CAAAGUGCUGUUCGUGCAGGUAG	[46,47]
hsa-miR-320a-3p	AAAAGCUGGGUUGAGAGGGCGA	[47,54,65]
hsa-miR-592	UUGUGUCAAUAUGCGAUGAUGU	[50]
hsa-miR-23a-3p	AUCACAUUGCCAGGGAUUUCC	[51]
hsa-miR-6767-5p	UCGCAGACAGGGACACAUGGAGA	[53]
hsa-miR-let-7b-3p	CUAUACAACCUACUGCCUUCCC	[62]
hsa-miR-1260a	AUCCCACCUCUGCCACCA	[62]
hsa-miR-424-5p	CAGCAGCAAUUCAUGUUUUGAA	[62]
hsa-miR-18b-5p	UAAGGUGCAUCUAGUGCAGUUAG	[62]

## Data Availability

Data generated or analyzed during this study are included in the published article.
